# Supplementary material for: Genome-wide enhancer-gene regulatory maps link causal variants to target genes underlying human cancer risk
Source: Nat Commun. 2023 Sep 25;14:5958. doi: 10.1038/s41467-023-41690-z (PMC10520073; doi:10.1038/s41467-023-41690-z)
Supplement: Supplementary file 1 — Supplementary Information [file 41467_2023_41690_MOESM1_ESM.pdf]

Supplementary Methods

Genomic annotation of ABC variants

For enrichment analysis, we first generated a set of control variants (non-ABC variants) with the allele frequencies, number of variants in LD, as well as genomic distribution matched to ABC variants for each cancer type using a web tool vSampler (http://mulinlab.org/vsampler/). SnpEff (v5.1) [1] was used to annotate the genomic annotation for both ABC variants and non-ABC variants. Variants would be classified into the following categories: upstream gene, downstream gene, 5'UTR, 3'UTR, intron, intergenic region, and other variants. Enrichment analyses of genomic annotation were performed by two-tailed Fisher's exact test with Bonferroni correction as the following 2 × 2 table (columns; ABC variants and non- ABC variants rows; SNPs within and not within the annotated genomic region).

Enrichment analyses of ABC variants among functional annotation

The functional annotation files for ChIP-seq peaks of histone modification including H3K4 monomethylation marks (H3K4me1), H3K4 dimethylation marks (H3K4me2), H3K4 trimethylation marks (H3K4me3), H3K27 acetylation marks (H3K27ac), H3K9 acetylation marks (H3K9ac), H3K27 trimethylation marks (H3K27me3), H3K36 trimethylation marks (H3K36me3) and TF-binding sites (TFBS) were downloaded from the ENCODE portal (https://www.encodeproject.org), and types of cell lines and tissues were provided in the **Supplementary Data 6**. BEDtools (v.2.27.1) was used to identify ABC variants or control variants overlapped with the peaks of regulatory elements. The core 15 chromatin state were downloaded from Roadmap (https://egg2.wustl.edu/roadmap/web_portal/chr_state_learning.html#core_15state). Enrichment analyses of ABC variants among regulatory elements were performed by two-tailed Fisher's exact test with the following 2×2 table (columns; ABC variants and non-ABC variants, rows; variants within and not within the regulatory element). We performed 59 individual TFs with 50,000 or more records of binding sites (each record is ~150 bp genomic region) in the bed file for the enrichment analyses of ABC variants, comparing with the random extracted non-ABC variants.

Enrichment analyses of ABC variants among cancer-related GWAS loci

We obtained the GWAS summary statistics for each cancer type from the GWAS catalog and selected SNPs that achieved genome-wide significance ($P < 5 \times 10^{-6}$) for subsequent enrichment analyses. GWAS loci was then defined as the genomic region encompassing SNPs in linkage disequilibrium (LD) with the index SNP at $r^2 \geq 0.2$. Enrichment analyses of ABC variants among cancer-related GWAS loci were performed by two-tailed Fisher's exact test (with the 2×2 table (columns; ABC variants and non-ABC variants, rows; variants within and not within the GWAS loci). Next, we estimated the heritability enrichment of ABC variants based on LD score regression (LDSC) by using GWAS summary statistics from previous study [2]. The enrichment for each annotation was calculated as the proportion of heritability over the proportion of SNPs within that

annotation category, and the standard error was estimated and used for *P* value calculation. GWAS SNPs that also belonged to ABC variants were extracted and used to plot the quantile-quantile plot (QQ plot) of the GWAS *P* values for those SNPs. Additionally, we obtained CRC GWAS summary statistics from a meta data of four large CRC consortia (the Colorectal Cancer Transdisciplinary (CORECT) Study, the Colon Cancer Family Registry (CFR), the Molecular Epidemiology of Colorectal Cancer (MECC) Study and the Genetics and Epidemiology of Colorectal Cancer Consortium (GECCO)) for analyze the role of ABC variants from CRC tissues in CRC risk variation.

Functional annotation of ABC enhancer target genes

To investigate the concrete pathways involved in ABC enhancer target genes (ABC genes), we downloaded 50 "hallmark" gene sets from the Molecular Signature Database (MSigDB) [3]. Genes that not regulated by ABC enhancers were considered as control genes (ABC score < 0.02). Pathway enrichment analyses was performed by using a two-tailed Fisher's exact test based on the hypergeometric distribution to test whether ABC genes are significantly enriched in the hallmark gene sets. The pathways with *P*-value < 0.05 was considered as significant enrichment.

To evaluate the genomic variation of ABC genes, we first downloaded the somatically mutated genes and somatic copy-number alterations (SCNAs) data for each cancer type were obtained from the TCGA data portal. The SCNAs of genes was visualized by the "maftools" package in R software. We also downloaded masked copy number segment files from TCGA data portal, and determined significant focal copy number alterations by GISTIC 2.0 [4]. For each locus, a sample is called deep amplification if the value is +2 (i.e., higher than the maximum of these arm values), while deep deletion is defined if the value is −2. Shallow (+/− 1) amplifications and deletions correspond to alterations between 0.1 relative copy number and the thresholds for deep alterations. Enrichment analyses of ABC genes with somatical mutation or copy number variation was performed by a two-tailed Fisher's exact test.

In addition, the gene expression profile and drug sensitivity data of human cancer cell lines were downloaded from the Genomics of Drug Sensitivity in Cancer (GDSC, (http://www.cancerrxgene.org/). This dataset provides IC50 values that indicate the sensitivity of the corresponding drug. The associations between the expression of ABC genes and drug sensitivity were evaluated by Spearman's rank correlation analysis. *P* value < 0.05 was considered as drug sensitivity associated. We further estimated the immune cell infiltration levels of each cancer type based on expression level of ABC genes. The infiltrates' abundances of six tumor-infiltrating immune cells computed by TIMER(B cells, $CD4^{+}$T cells, $CD8^{+}$T cells, macrophages, neutrophils, and dendritic cells) were downloaded from Tumor Immune Estimation Resource (TIMER 2.0) (http://timer.cistrome.org/). The associations between ABC gene expression and immune infiltrates were evaluated by Spearman's rank correlation analysis and *P* < 0.05 was defined as immune

infiltrates-related. The proportions of immune-related ABC genes are the number of ABC genes correlated with immune infiltrates divided by the total number of ABC genes.

HiC data processing

We downloaded HiC raw sequenced data of 4 transverse colon from ENCODE project, including ENCSR295BDK, ENCSR504OTV, ENCSR079IDJ and ENCSR424WMG. The contact matrices to 5-kb resolution from restriction fragments were processed and normalized by Knight and Ruiz (KR) normalization with Juicer (v1.7.5, https://github.com/aidenlab/juicer). We subsequently merged the contacts across 4 biosamples to get the average chromatin interaction frequency and scaled the HiC signal using the power-law distribution.

Comparisons ABC model to previous enhancer-gene predictions

We compared ABC model to methods using alternative enhancer-gene linking approaches. First, we browsed the published literatures on regulatory variants of CRC from PubMed, and sorted out a credible set consisting of 27 variant-gene connections which were validated by functional experiments (**Supplementary Table 2**). We used the predictions from each method to overlap variants with enhancers and assigned genes to the regulatory variants. For the method based on genome position, we obtained the closet gene for each variant in Haploreg and mapped variants to genes using MAGMA [5]. For eQTL methods, we analyzed the genes that were statistically associated with variants using GTEx and eQTLGen datasets, as well as previously published eQTL results in colon tissues [6]. For the method based on 3D loops, we downloaded pc-HiC data in colon tissue from 3DIV dataset and selected the promoter-other region pairs. Meanwhile, we downloaded the related file from previous studies that reported enhancer-gene predictions, including linking distal accessible elements with gene promoters by looking at correlation of DNase I hypersensitivity from ENCODE from a previous study [7], linking gene expression with the enhancers (regions with the '7Enh' ChromHMM state) which were predicted from five active chromatin marks (H3K27ac, H3K9ac, H3K4me1, H3K4me2 and DNase I hypersensitivity) using the Roadmap epigenome atlas [8], and linking transcriptional activity of enhancers and TSSs using the FANTOM5 CAGE expression atlas [9].

Supplementary Figures

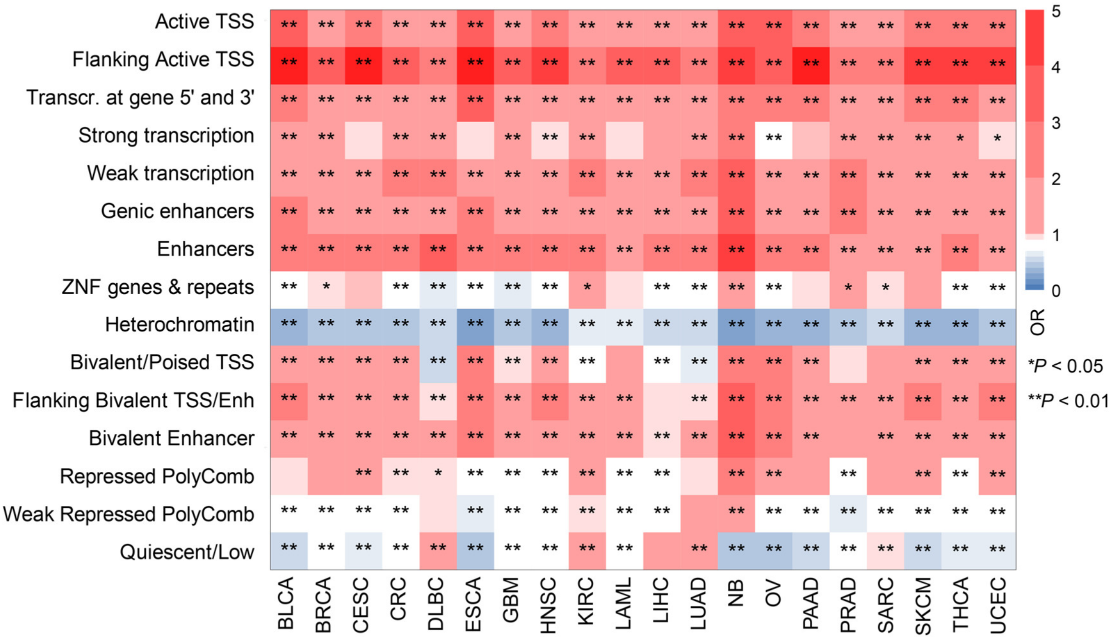

Figure S1. Enrichment analyses of ABC variants in each functional category of chromatin state compared with non-ABC variants. The core 15 chromatin state were downloaded from Roadmap. *P* values were calculated by two-tailed Fisher's exact test. Source data are provided with this paper.

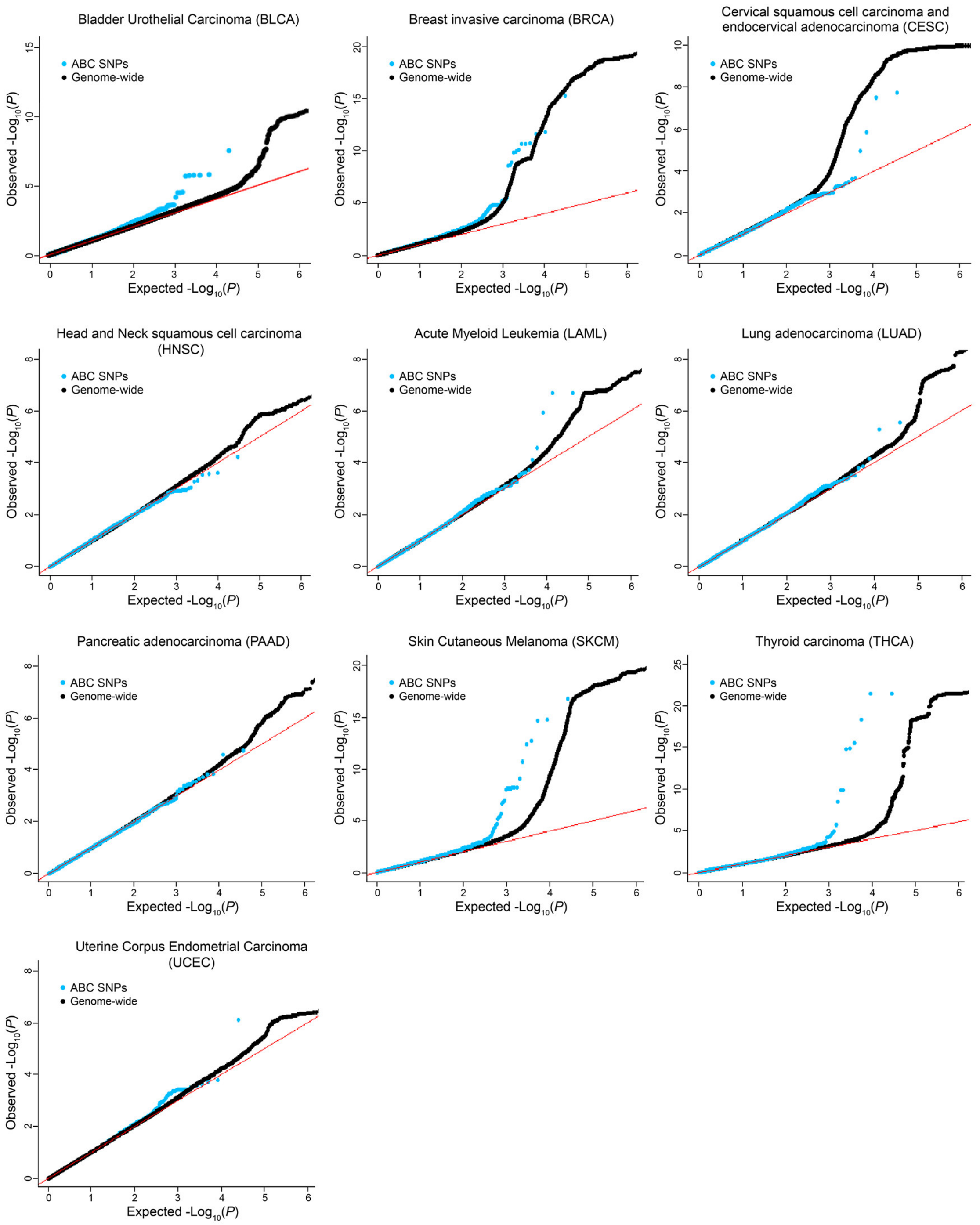

Figure S2. Quantile-quantile (QQ) plots of *P* values from GWAS among multiple cancer types. ABC variants were shown in comparison with genome wide variants. GWAS variants were binary annotated using ABC variants with $P < 0.05$.

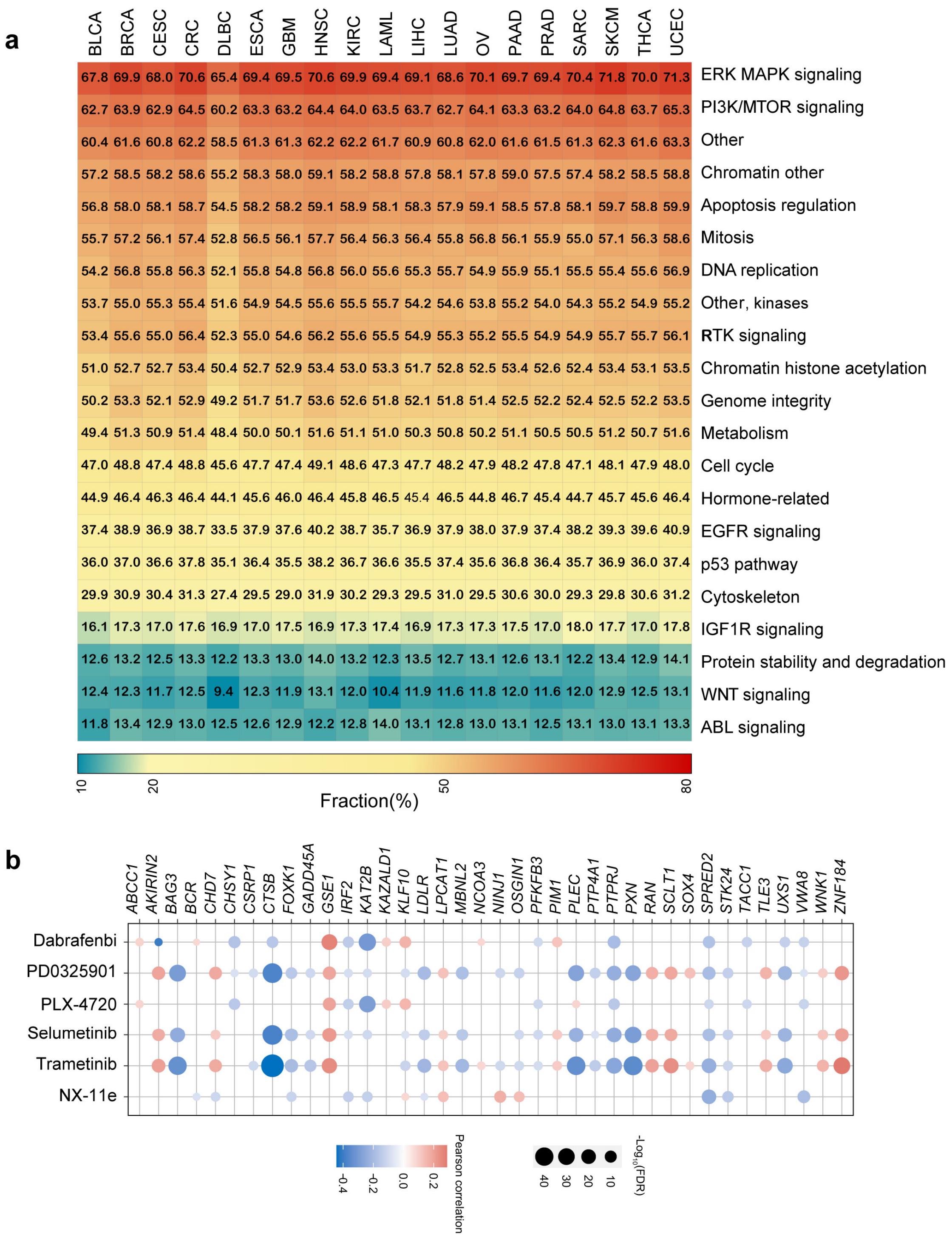

Figure S3. Summary of drug response for ABC target genes. a. The association between ABC genes expression level and drug response based on the GDSC drug dataset. Values in the heatmap represented the fractions of significantly associated ABC gene-drug pairs. **b.** An example for the association of ABC genes expression with drug sensitivity in the drugs that target ERK/MAPK signaling. The circle color indicates the Pearson correlation, and circle size indicates false discovery rate (FDR). Source data are provided with this paper.

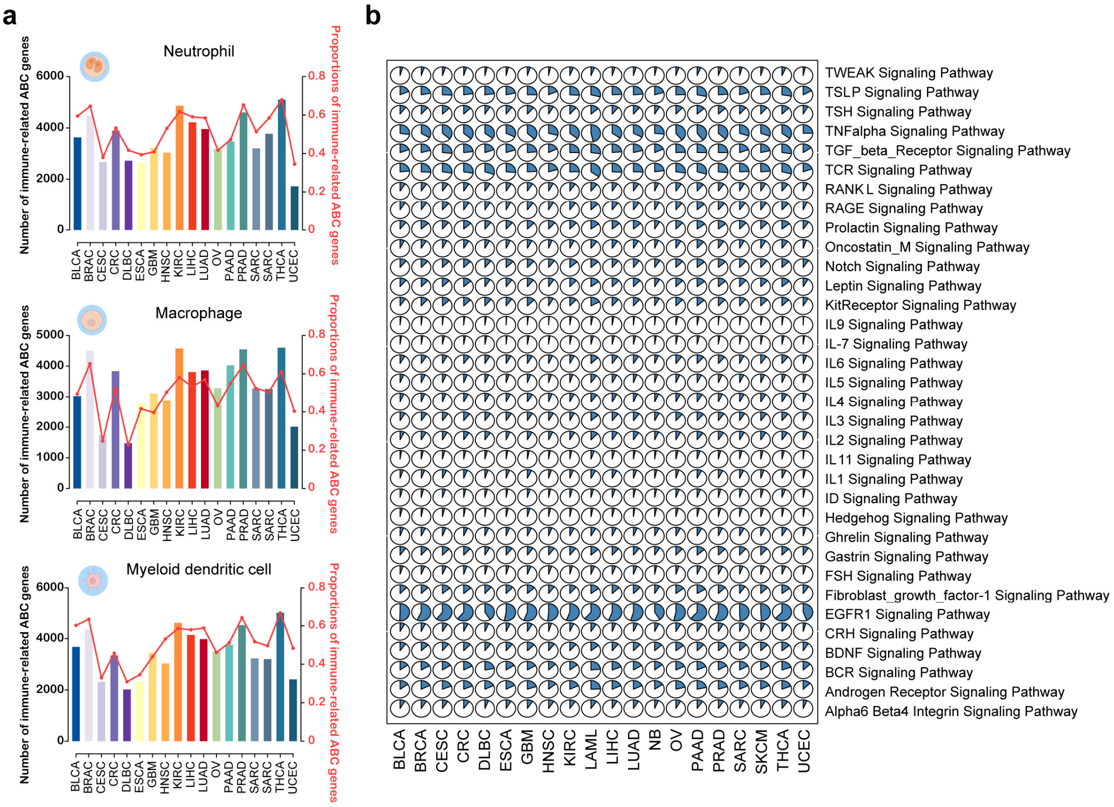

Figure S4. Summary of immune response for ABC target genes. a. The association between the expression of ABC genes and infiltration proportion of immune cells (Neutrophil, Macrophage, Myeloid dendritic cell) that was estimated by TIMER. The right y-axis represented the number of ABC genes associated with immune cell infiltration and the right y-axis represented the proportion of these immune-related ABC genes. The cell symbols were created with BioRender.com. **b.** Scatterpie chart of the proportion of ABC genes enriched in immune-related pathways based on GSEA. Source data are provided with this paper.

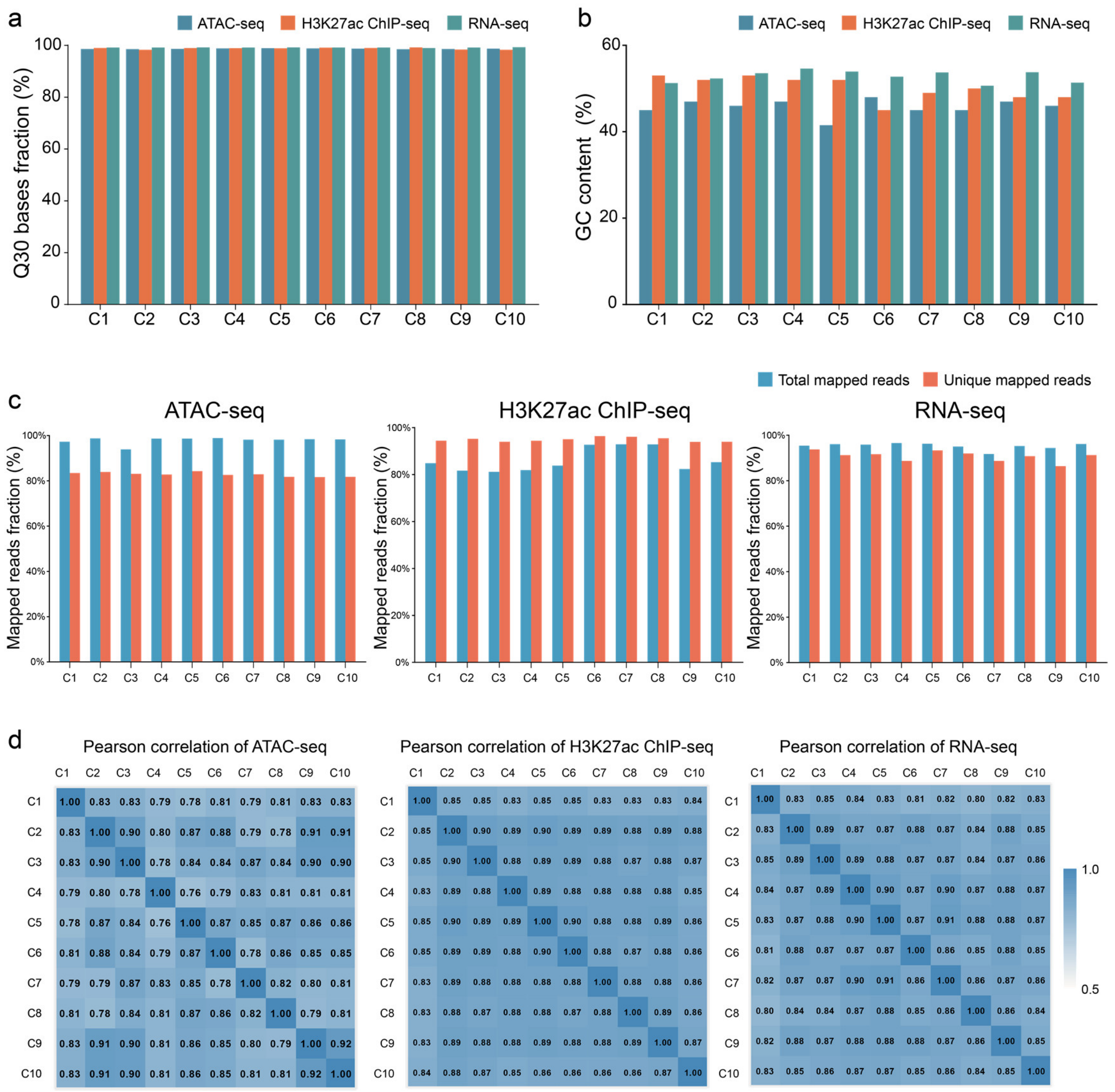

Figure S5. Quality assessment of the high-throughput sequence data in our CRC tissues. a. The fraction of the sequenced bases with a quality score of at least Q30. **b.** The fraction of the GC content. **c.** The fraction of the reads that mapped to human reference genome. **d**. Pearson correlation analysis of sequence data between 10 CRC samples. The Pearson correlation analysis was conducted using deeptools. Source data are provided with this paper.

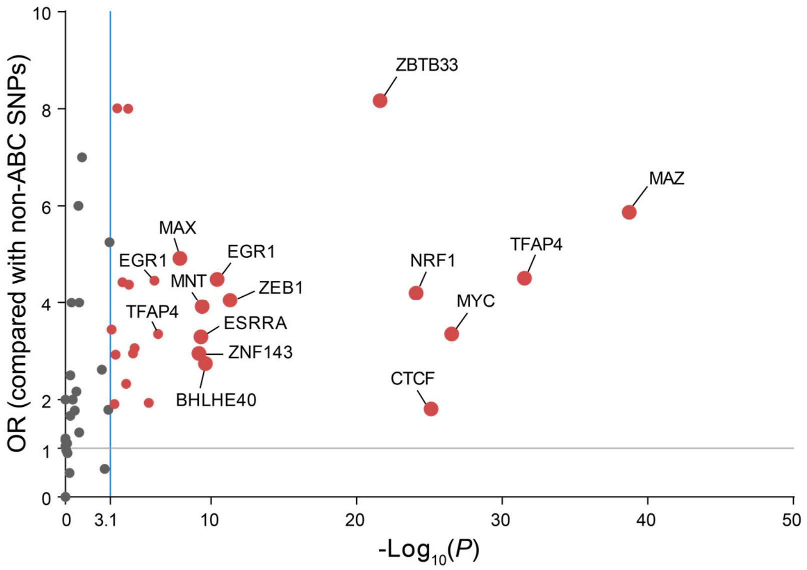

Figure S6. Enrichment of ABC variants within TF-binding sites. Plots of -log$_{10}$ P-values (x-axis) and OR (y axis) obtained from enrichment analysis of ABC variants among variants within binding sites for each TF. The blue line indicates $P = 0.05/59 = 8.47 \times 10^{-4}$ (Bonferroni-corrected P-value threshold, binding sites for a total of 59 TF were tested). Enrichment analysis were calculated by two-tailed Fisher's exact test. Source data are provided with this paper.

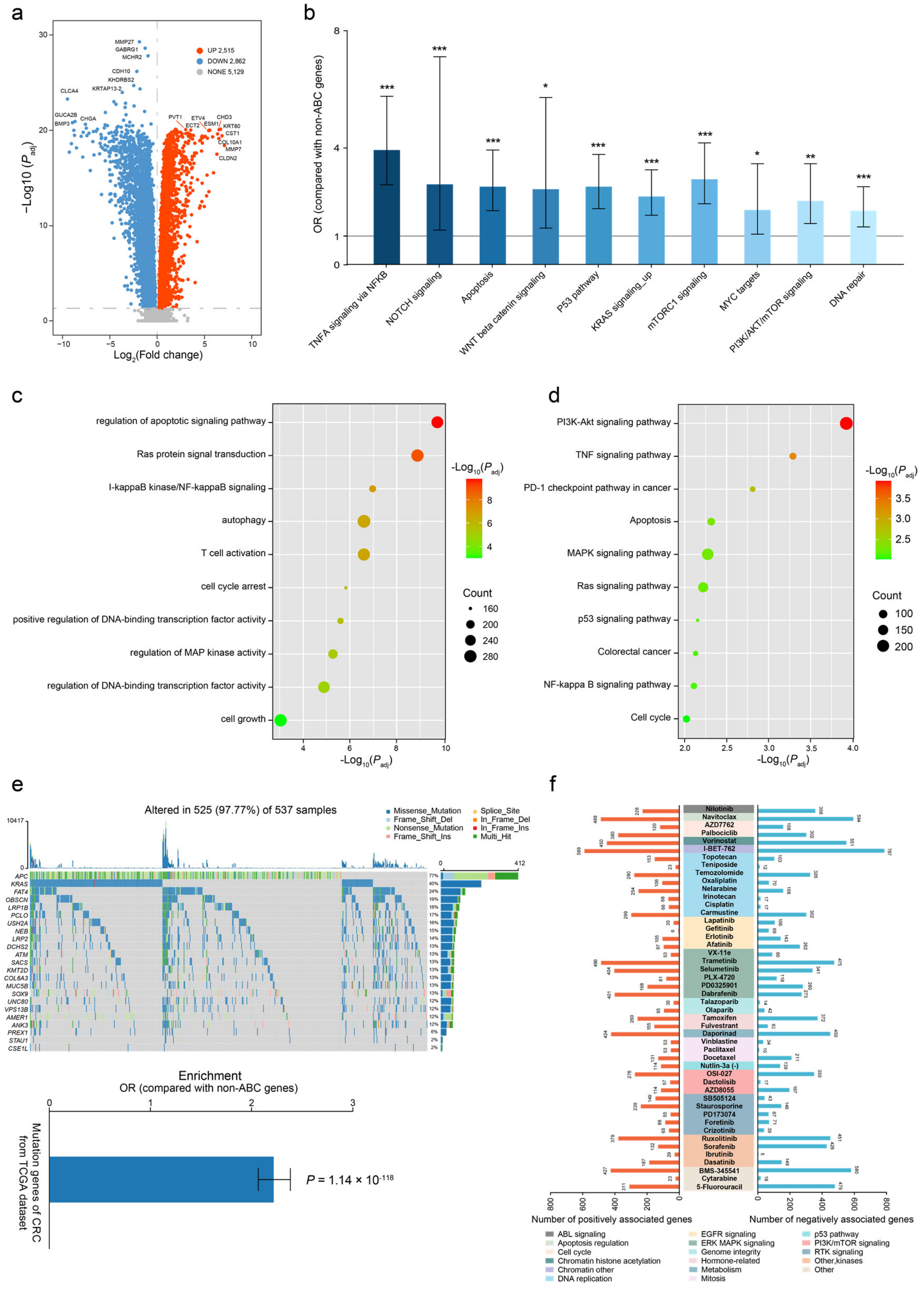

Figure S7. Functional characterization of ABC target genes identified from our CRC tissues. a. Differentially expressed ABC genes in TCGA dataset. Differential expression analysis was performed from read counts using DESeq2 package in R with default parameters. $P_{adj} < 0.05$ were used to obtain a final list of different expression genes. **b.** The enrichment of differentially expressed ABC genes in MsigDB hallmark gene sets. The bars indicated enrichment value with 95% CIs,

***$P < 0.0001$, **$P < 0.001$, *$P < 0.01$ were calculated by a two-sided Student's t-test. **c, d.** GO enrichment **(c)** and KEGG pathways enrichment **(d)** of differentially expressed ABC genes. **e.** Somatic mutation landscape for ABC genes among TCGA CRC samples. The waterfall plot showed top 20 ABC genes mutation for every sample and mutation subtypes. The bar chart indicated the enrichment of ABC genes in somatically mutated genes set compared with non-ABC genes with 95% CI. **f.** The identified ABC genes-drug association pairs based on GDSC dataset. Blue bar denoted negative association and red bar denoted positive association, respectively. Source data are provided with this paper.

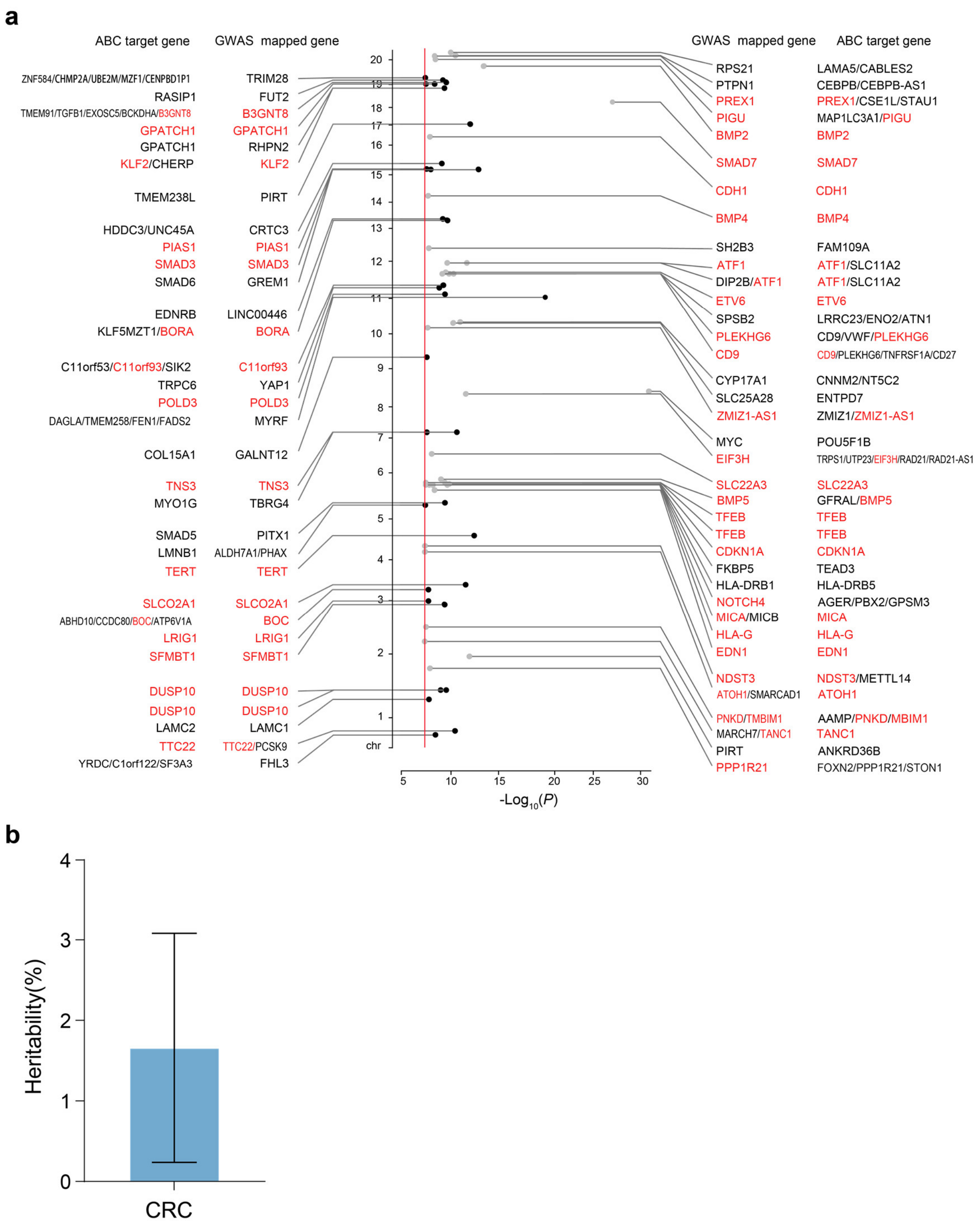

Figure S8. Enrichment of ABC variants identified from our CRC tissues among GWAS loci. **a.** ABC variants covered 69 CRC GWAS loci linking to 111 genes. Each dot represented the tag SNP of each GWAS locus that was overlapped with ABC variants. The target genes predicted by ABC model in each locus that consistent with GWAS mapped genes were marked in red (39 genes). The P values of tag variants were summarized from previous GWAS and the red line indicated $P = 5\times10^{-8}$ threshold. **b.** Proportion of GWAS trait heritability of CRC explained by ABC variants. The error bars represented standard error.

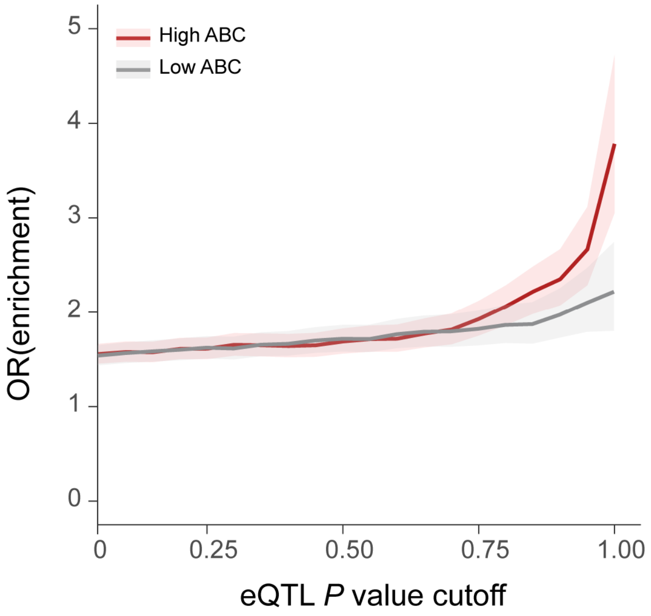

Figure S9. Enrichment for ABC variants identified from our CRC tissues also being TCGA eQTL variants compared to non-ABC variants. Enrichment are shown over a range of eQTL *P* value in descending order cutoffs. Line indicate the ORs and the shaded areas represent 95% confidence intervals (CI). Source data are provided with this paper.

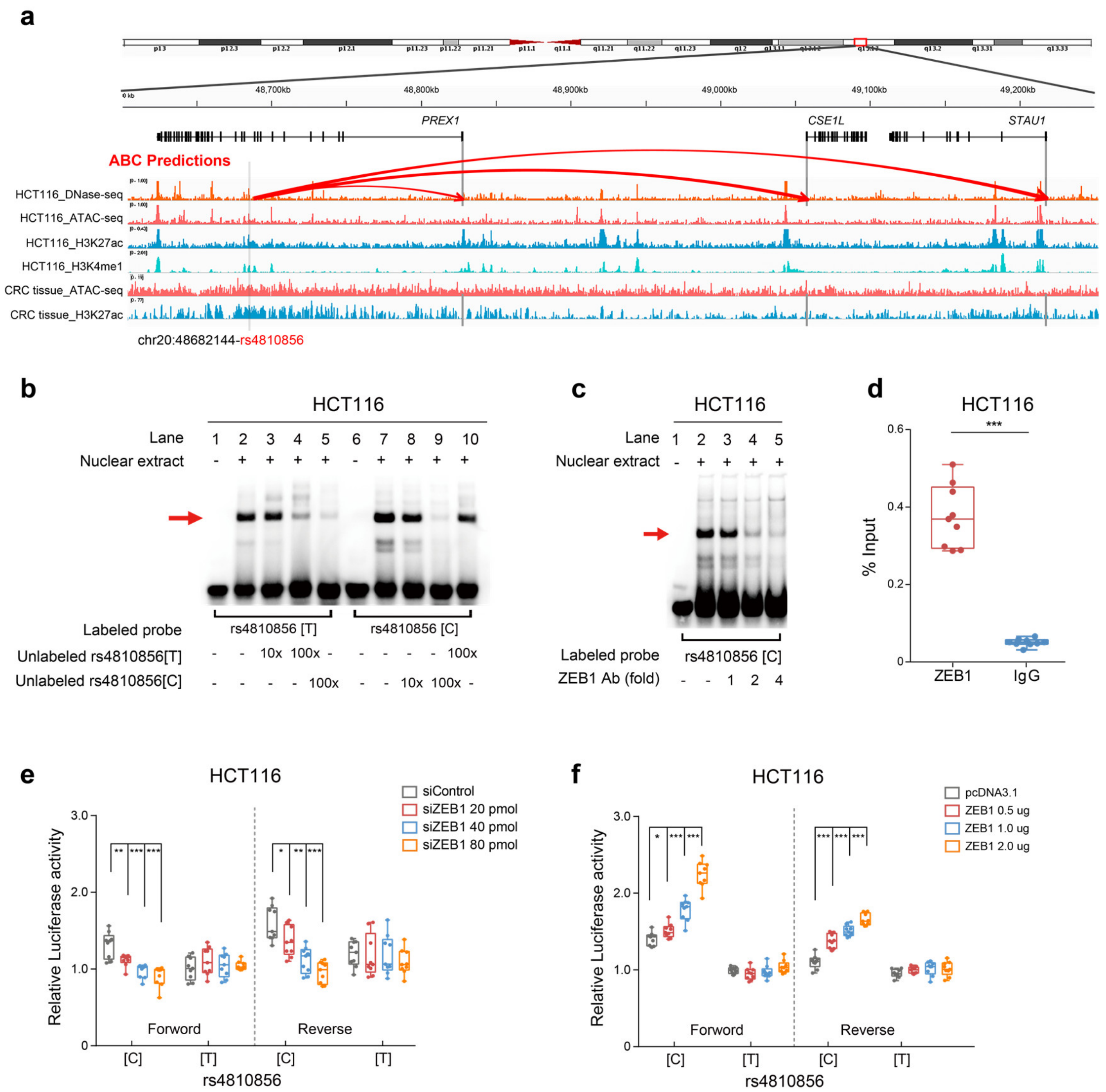

Figure S10. ABC variant rs4810856 acts as an allele-specific enhancer to regulate *PREX1, CSE1L* and *STAU1* expression. a. Epigenetic annotation for the region surrounding rs4810856 in CRC cell lines and tissues. The epigenetic tracks of HCT116 cells including DNase-seq, ATAC-seq peaks, multiple histone (H3K27ac, H3K4me1) modification peaks, and ChIP-seq peaks for ZEB1 were obtained from the Cistrome database. The epigenetic tracks of CRC tissues (ATAC-seq and H3K27ac modification peaks) were obtained from 10 CRC biosamples and visualized by the Integrative Genomics Viewer (IGV). **b.** EMSAs with biotin-labeled probes containing rs4810856 in HCT116 cells. Arrows indicated allele-specific bands that interact with nuclear protein in the cells. 10× and 100× represented 10-fold and 100-fold excess amounts of an unlabeled probe compared with the amount of the labeled probe. "+" and "–" indicate added and not added, respectively. **c.** ZEB1 super-shift EMSAs with biotin-labeled probes containing rs4810856 in HCT116 cells. 1×, 2× and 4× represented 0.1μg, 0.2μg and 0.4μg of ZEB1 antibody. **d.** Chromatin enrichment of ZEB1 at the rs4810856 site measured by ChIP-qPCR assays in HCT116 cells. Data were normalized to the input from three repeated experiments, each with three replicates. The center line of the box presentation as the median, box limits indicated upper and lower quartiles, and whiskers indicated

the maximum and minimum values. IgG served as negative control. ****P* < 0.0001 were calculated by a two-sided Student's *t*-test. **e-f.** The effect of *ZEB1* knockdown **(e)** and overexpression **(f)** on relative luciferase activity of vectors containing rs4810856 [C] or rs4810856 [T] allele in HCT116 cells. The center line of the box presentation as the median, box limits indicated upper and lower quartiles, and whiskers indicated the maximum and minimum values. ****P* < 0.0001, ***P* < 0.001, **P* < 0.01 were calculated by a two-sided Student's t-test, from three independent experiments with three technical replicates. Source data are provided with this paper.

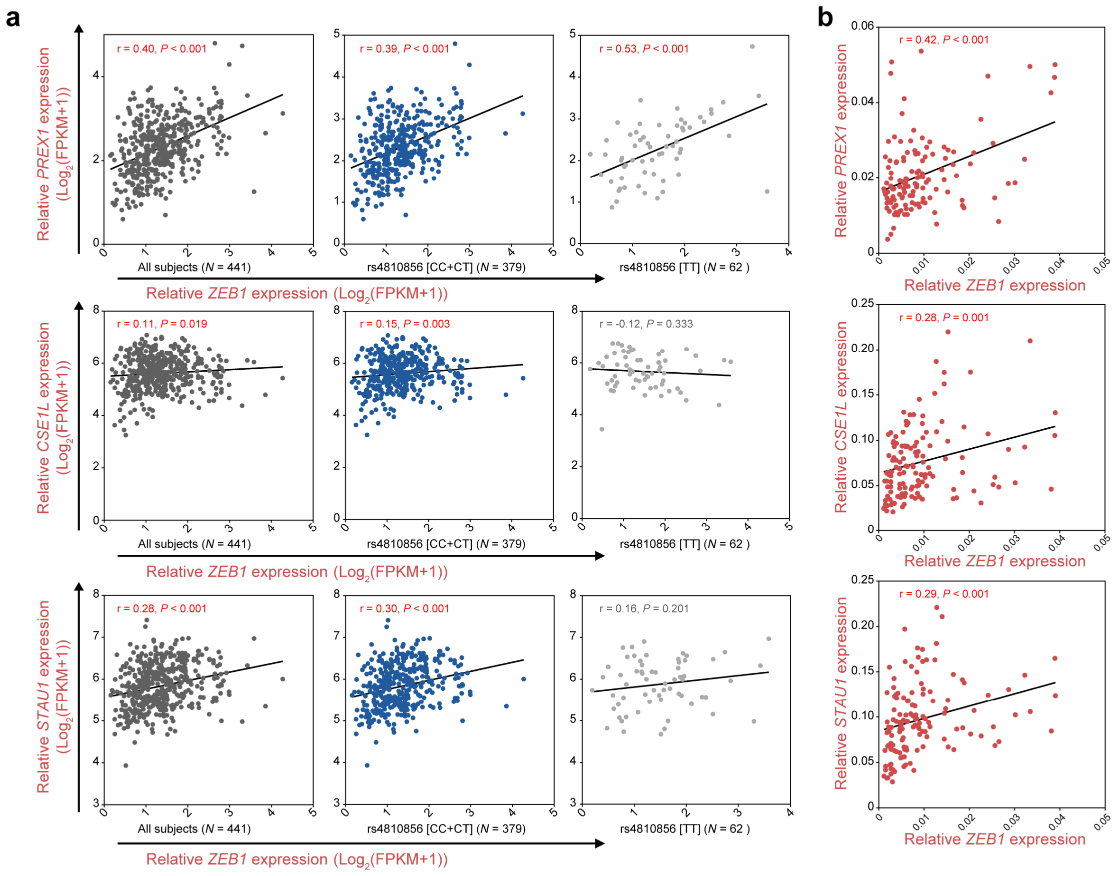

Figure S11. The correlations of *ZEB1* expression with *PREX1*, *CSE1L* and *STAU1* expression were analyzed in TCGA cohort (a) and our own CRC patients (b). All *P* values and *r* values were calculated by Pearson's correlation analysis.

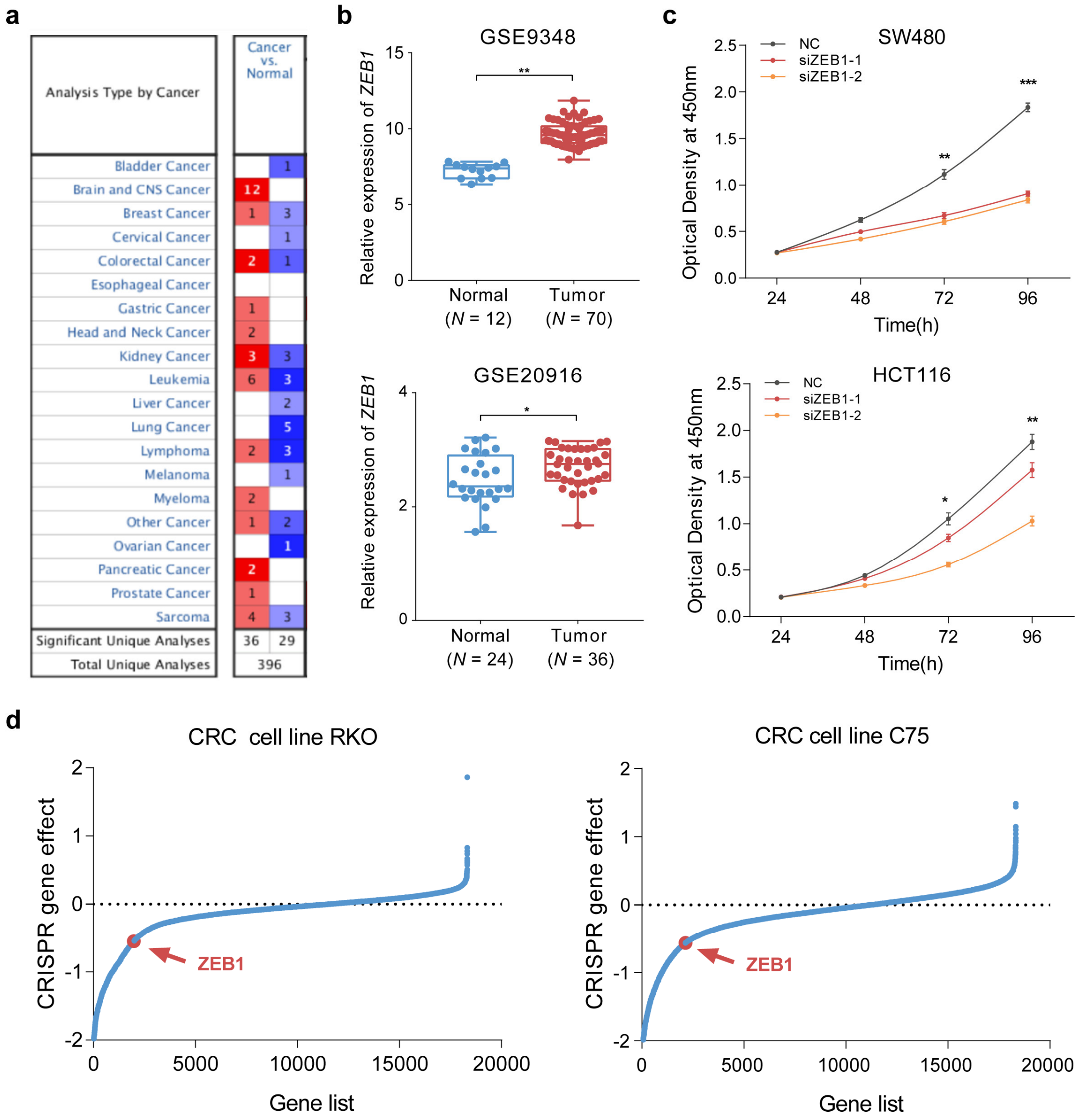

Figure S12. Transcriptional factor ZEB1 can function as a potential oncogene in CRC. a. *ZEB1* expression levels were evaluated in various tumor tissue types from Oncomine database. **b.** Expression levels of *ZEB1* in CRC tissues and adjacent normal tissues from two GEO datasets (GSE9348 and GSE20916). The center line of the box presentation as the median, box limits indicated upper and lower quartiles, and whiskers indicated the maximum and minimum values. ***P* <0.001, **P* <0.01 were calculated by a two-sided Student's *t*-test. Error bars represented mean ± SEM. **c.** The effect of *ZEB1* knockdown on cell proliferation in SW480 and HCT116 cells. Results were shown as the median (minimum to maximum) from triplicate experiments. ****P* < 0.0001, ***P* < 0.001 were calculated by a two-sided Student's *t*-test. **d.** The potential effect of *ZEB1* in CRC cell lines RKO and C75 from the data of genome-wide CRISPR/Cas9-based loss-of-function screens. Source data are provided with this paper.

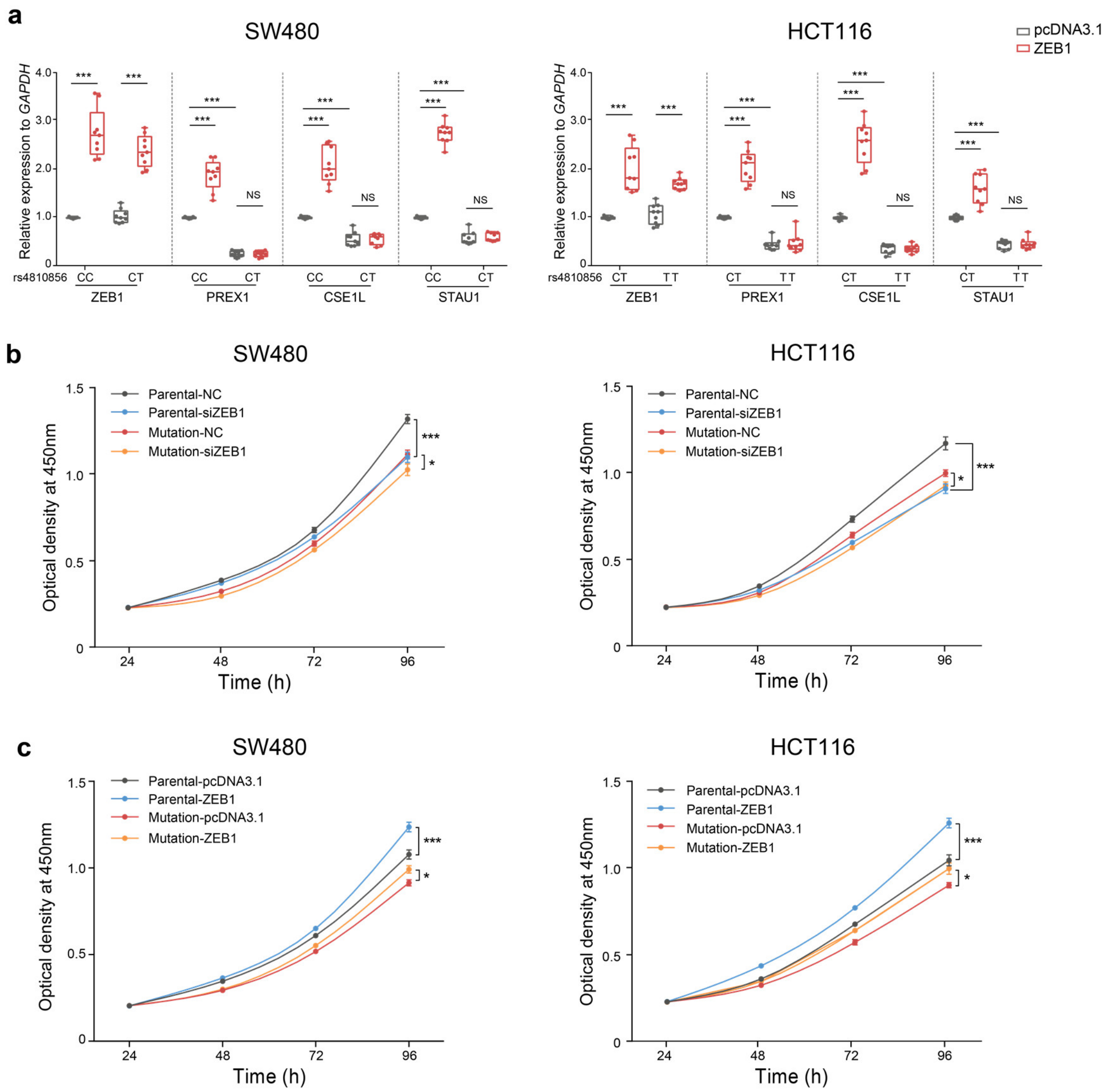

Figure S13. Direct effects of rs4810856 on target genes expression and cell proliferation. a. The effects of *ZEB1* overexpression on *PREX1, CSE1L* and *STAU1* expression in parental (SW480[CC] and HCT116[CT]) and mutated cells (SW480[CT] and HCT116[TT]). The center line of the box presentation as the median, box limits indicated upper and lower quartiles, and whiskers indicated the maximum and minimum values. ***P < 0.0001, **P < 0.001, *P < 0.01 were calculated by a two-sided Student's t-test, from three independent experiments with three technical replicates. **b-c.** The direct effect of rs4810856 genotype on cell proliferation upon ZEB1 knockdown **(b)** or overexpression **(c)**. Results were shown as the means ± SEM from triplicate experiments. ***P < 0.0001, *P < 0.01 were calculated by a two-sided Student's *t*-test. Source data are provided with this paper. Source data are provided with this paper.

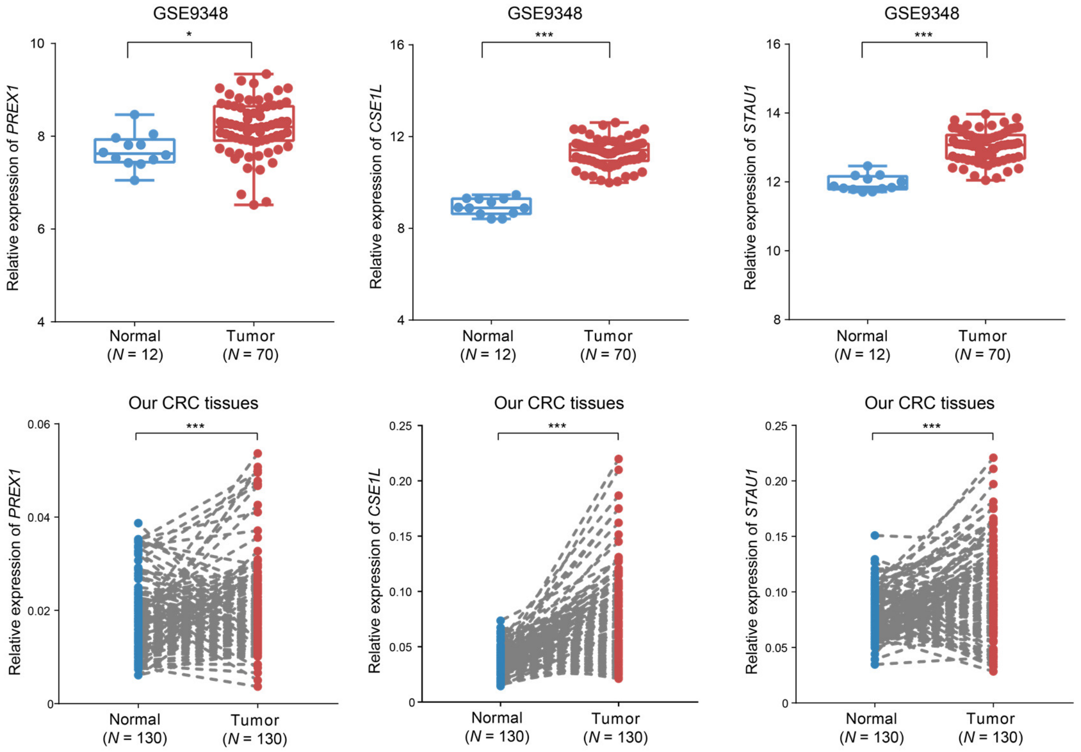

Figure S14. *PREX1*, *CSE1L* and *STAU1* function as oncogenes in CRC. Expression levels of *ZEB1* in CRC tissues and adjacent normal tissues in GSE9348 datasets and our own CRC tissues. The center line of the box presentation as the median, box limits indicated upper and lower quartiles, and whiskers indicated the maximum and minimum values. ***P <0.0001, *P <0.01 were calculated by a two-sided Student's t-test. Error bars represented mean ± SEM.

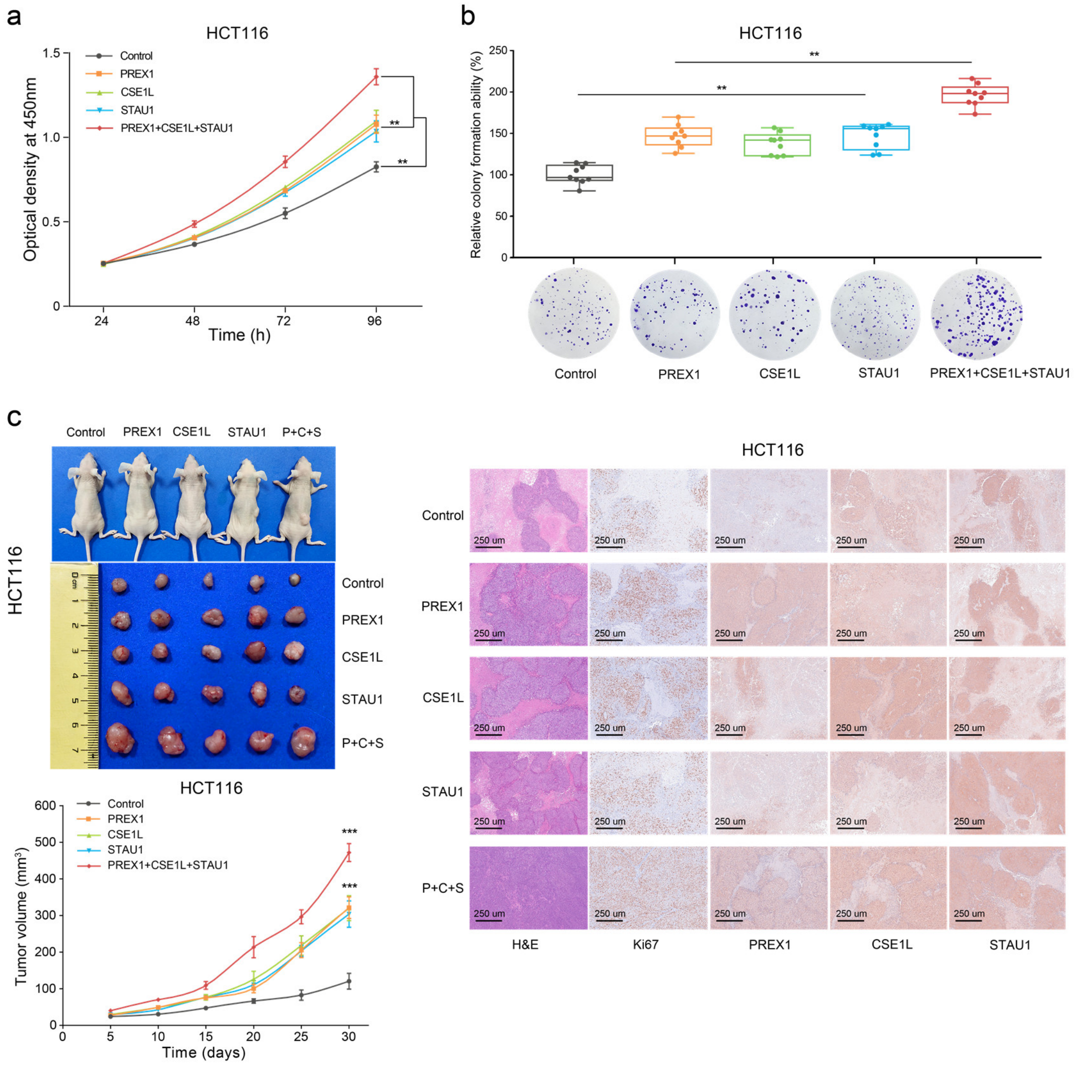

Figure S15. *PREX1*, *CSE1L* and *STAU1* exert synergistic effects to promote CRC development. **a.** The effect of *PREX1*, *CSE1L* and *STAU1* on cell proliferation in lentivirus-mediated HCT116 cells. Results were shown as the median (minimum to maximum) from triplicate experiments. ***$P < 0.0001$, **$P < 0.001$ were calculated by a two-sided Student's *t*-test. **b.** The effect of *PREX1*, *CSE1L* and *STAU1*on colony formation ability in lentivirus-mediated HCT116 cells. The results presented colony formation ability relative to control cells (set to 100%). Data were shown as the median (minimum to maximum) from triplicate experiments. The center line of the box presentation as the median, box limits indicated upper and lower quartiles, and whiskers indicated the maximum and minimum values. **$P < 0.001$were calculated by a two-sided Student's *t*-test. **c.** The effect of *PREX1*, *CSE1L* and *STAU1* on CRC tumor growth *in vivo*. Representative images, growth curves of xenograft tumors, representative H&E staining and immunohistochemical analysis (Ki67, PREX1, CSE1L and STAU1) derived from lentivirus-mediated HCT116 cells were shown. The results were shown as the means ± SD for five mice per group. ***$P < 0.0001$ were calculated by a two-sided Student's *t*-test. Source data are provided with this paper.

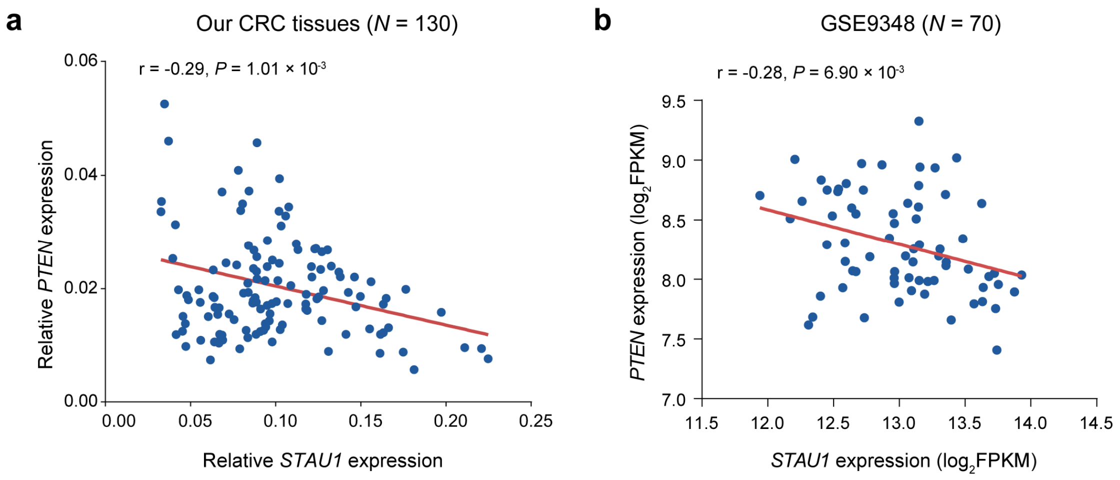

Figure S16. The correlation of *STAU1* expression with *PTEN* expression. The correlations of *STAU1* expression with *PTEN* expression were analyzed in our own CRC patients (**a**) and GEO dataset (**b**). *P* value and *r* value were calculated by Pearson's correlation analysis.

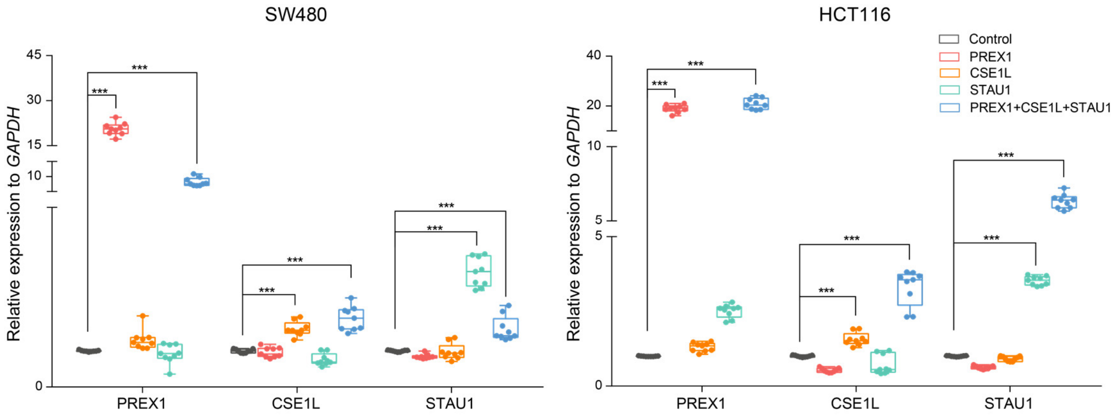

Figure S17. The expression level of *PREX1*, *CSE1L* and *STAU1* on lentivirus-mediated overexpression in SW480 and HCT116 cells. The center line of the box presentation as the median, box limits indicated upper and lower quartiles, and whiskers indicated the maximum and minimum values. ***P < 0.0001 were calculated by a two-sided Student's t-test, from three independent experiments with three technical replicates.

Supplementary Tables

Supplementary Table 1. The clinical characteristics of the 10 CRC patients

Sample ID	Gender	Age	Tumor location	Tumor pathology	TNM
C1	Male	73	Colon	Moderately and poorly differentiated adenocarcinoma	T3N1bM0
C2	Male	53	Sigmoid colon	Moderately differentiated adenocarcinoma	pT3N0Mx
C3	Male	54	Rectum	Moderately differentiated adenocarcinoma	pT3N0Mx
C4	Male	54	Rectum	Moderately differentiated adenocarcinoma	pT2N0Mx
C5	Male	61	Rectum	Moderately differentiated adenocarcinoma	pT2N1cMx
C6	Female	56	Sigmoid colon	Moderately differentiated adenocarcinoma	pT3N1bMx
C7	Female	72	Ascending colon	Poorly differentiated adenocarcinoma	pT3N1M0
C8	Female	53	Rectum	Moderately and poorly differentiated adenocarcinoma	pT2N2M0
C9	Female	50	Sigmoid colon	Moderately differentiated adenocarcinoma	pT3N0Mx
C10	Female	50	Rectum	Moderately and poorly differentiated adenocarcinoma	pT4aN0Mx

Supplementary Table 2. Credible set of SNP-gene connections validated by functional experiments

Chr	Pos (GRCh38)	SNP	Target Gene	Reference (PMID)
15	32717277	rs1406389	*GREM1*	33476087
12	50775325	rs7959129	*ATF1*	31204011
8	127401060	rs6983267	*MYC*	19561604
11	111283347	rs7130173	*C11orf53*	24256810
11	111283347	rs7130173	*C11orf92*	24256810
11	111283347	rs7130173	*C11orf93*	24256810
12	6272341	rs11064124	*CD9*	31988071
12	6272341	rs11064124	*PLEKHG6*	31988071
8	143813383	rs13251492	*SCRIB*	33011827
12	11856807	rs2238126	*ETV6*	27145994
7	148827660	rs3757441	*EZH2*	26553291
11	61834531	rs174575	*FADS2*	32127356
11	61834531	rs174575	*AP002754.2*	32127356
6	160387116	rs420038	*SLC22A3*	30561001
8	1979843	rs11777210	*KBTBD11*	29267898
5	135141069	rs17716310	*SMAD5*	27177089
3	47281291	rs1076394	*CCDC12*	27120998
3	47281291	rs1076394	*NME6*	27120998
8	127894542	rs7017386	*PVT1*	34274452
8	127894542	rs7017386	*CCAT1*	34274452
5	132101268	rs27437	*SLC22A5*	29428571
16	68784487	rs7198799	*ZFP90*	31641208
5	130990246	rs1010208	*HINT1*	33123275
18	48922435	rs6507874	*SMAD7*	25375357
18	48922449	rs6507875	*SMAD7*	25375357
18	48922741	rs8085824	*SMAD7*	25375357
18	48923195	rs58920878	*SMAD7*	25375357

Supplementary Table 3. Characteristics of the GECCO population

Variable	Cases (*N* = 17,789)<br>*N* (%)	Controls (*N* = 19,951)<br>*N* (%)	*P*
**Gender**			<0.001*
Male	10,128 (56.93)	10,298 (51.62)	
Female	7,66 (43.07)	9,653 (48.38)	
**Age** (mean ± SD, years)	63.83 ± 10.31	59.97 ± 12.32	<0.001†
**Age groups** (years)			<0.001*
≤ 49	1,490 (8.38)	3,485 (17.47)	
50-59	4,470 (25.13)	5,497 (27.55)	
60-69	6,498 (36.53)	6,612 (33.14)	
≥70	5,331 (29.97)	4,357 (21.84)	

**P* value was calculated by Pearson χ^2 test.

†*P* value was calculated by a two-sided Student's *t*-test.

Supplementary References

1. Cingolani P, *et al.* A program for annotating and predicting the effects of single nucleotide polymorphisms, SnpEff: SNPs in the genome of Drosophila melanogaster strain w1118; iso-2; iso-3. *Fly (Austin)* **6**, 80-92 (2012).

2. Rashkin SR, *et al.* Pan-cancer study detects genetic risk variants and shared genetic basis in two large cohorts. *Nature communications* **11**, 4423 (2020).

3. Liberzon A, Birger C, Thorvaldsdóttir H, Ghandi M, Mesirov JP, Tamayo P. The Molecular Signatures Database (MSigDB) hallmark gene set collection. *Cell systems* **1**, 417-425 (2015).

4. Mermel CH, Schumacher SE, Hill B, Meyerson ML, Beroukhim R, Getz G. GISTIC2.0 facilitates sensitive and confident localization of the targets of focal somatic copy-number alteration in human cancers. *Genome biology* **12**, R41 (2011).

5. de Leeuw CA, Mooij JM, Heskes T, Posthuma D. MAGMA: generalized gene-set analysis of GWAS data. *PLoS computational biology* **11**, e1004219 (2015).

6. Moreno V, *et al.* Colon-specific eQTL analysis to inform on functional SNPs. *British journal of cancer* **119**, 971-977 (2018).

7. Sheffield NC, *et al.* Patterns of regulatory activity across diverse human cell types predict tissue identity, transcription factor binding, and long-range interactions. *Genome research* **23**, 777-788 (2013).

8. Liu Y, Sarkar A, Kheradpour P, Ernst J, Kellis M. Evidence of reduced recombination rate in human regulatory domains. *Genome biology* **18**, 193 (2017).

9. Gao T, Qian J. EnhancerAtlas 2.0: an updated resource with enhancer annotation in 586 tissue/cell types across nine species. *Nucleic acids research* **48**, D58-d64 (2020).
