# Supplementary material for: Genome-wide enhancer-gene regulatory maps link causal variants to target genes underlying human cancer risk
Source: Nat Commun. 2023 Sep 25;14:5958. doi: 10.1038/s41467-023-41690-z (PMC10520073; doi:10.1038/s41467-023-41690-z)
Supplement: Supplementary file 3 — Description of Additional Supplementary Files [file 41467_2023_41690_MOESM3_ESM.pdf]

Description of Additional Supplementary Files

Supplementary Data 1. Summary of ABC maps in 20 cancer types.

Supplementary Data 2. Association analyses between enhancer variant rs4810856 and CRC risk in the public datasets.

Supplementary Data 3. Association analyses between ABC variant rs4810856 and CRC risk in the individuals from UK Biobank and GECCO.

Supplementary Data 4. STAU1 RNA-binding events identified by RIP-seq analysis.

Supplementary Data 5. Probes or primers sequence used in the study.

Supplementary Data 6. Summary of cancer cell lines used for function annotation
