# Supplementary material for: Genome-wide enhancer-gene regulatory maps link causal variants to target genes underlying human cancer risk
Source: Nat Commun. 2023 Sep 25;14:5958. doi: 10.1038/s41467-023-41690-z (PMC10520073; doi:10.1038/s41467-023-41690-z)
Supplement: Supplementary file 10 — Reporting Summary [file 41467_2023_41690_MOESM10_ESM.pdf]

nature portfolio

Corresponding author(s): Jianbo Tian Xiaoping Miao

Last updated by author(s): Sep 11, 2023

Reporting Summary

Nature Portfolio wishes to improve the reproducibility of the work that we publish. This form provides structure for consistency and transparency in reporting. For further information on Nature Portfolio policies, see our Editorial Policies and the Editorial Policy Checklist.

Statistics

For all statistical analyses, confirm that the following items are present in the figure legend, table legend, main text, or Methods section.

n/a	Confirmed	
☐	☒	The exact sample size (*n*) for each experimental group/condition, given as a discrete number and unit of measurement
☐	☒	A statement on whether measurements were taken from distinct samples or whether the same sample was measured repeatedly
☐	☒	The statistical test(s) used AND whether they are one- or two-sided<br>*Only common tests should be described solely by name; describe more complex techniques in the Methods section.*
☐	☒	A description of all covariates tested
☐	☒	A description of any assumptions or corrections, such as tests of normality and adjustment for multiple comparisons
☐	☒	A full description of the statistical parameters including central tendency (e.g. means) or other basic estimates (e.g. regression coefficient) AND variation (e.g. standard deviation) or associated estimates of uncertainty (e.g. confidence intervals)
☐	☒	For null hypothesis testing, the test statistic (e.g. *F*, *t*, *r*) with confidence intervals, effect sizes, degrees of freedom and *P* value noted<br>*Give P values as exact values whenever suitable.*
☒	☐	For Bayesian analysis, information on the choice of priors and Markov chain Monte Carlo settings
☒	☐	For hierarchical and complex designs, identification of the appropriate level for tests and full reporting of outcomes
☐	☒	Estimates of effect sizes (e.g. Cohen's *d*, Pearson's *r*), indicating how they were calculated

Our web collection on statistics for biologists contains articles on many of the points above.

Software and code

Policy information about availability of computer code

Data collection	The sequencing data of DNase-seq, ATAC-seq, H3K27ac ChIP-seq and Hi-C from GEO datasets was downloaded using prefetch in the SRA toolkit. The genotype data of GECCO cohort was downloaded using prefetch in the SRA toolkit. The genotype data of UK Biobank was downloaded using gfetch.
Data analysis	Computer code relating to this study includes:<br>ABC model: https://github.com/broadinstitute/ABC-Enhancer-Gene-Prediction<br>Python v.3.7.0: https://www.python.org/downloads/release/python-364/<br>Trimmomatic v.0.36: https://github.com/usadellab/Trimmomatic<br>FastUniq v.1.1: https://anaconda.org/bioconda/fastuniq<br>bowtie2 v.2.2.6: https://github.com/BenLangmead/bowtie2/releases<br>samtools v.1.12: https://sourceforge.net/projects/samtools/files/samtools/0.1.19/<br>bedtools v.2.27.1: https://sourceforge.net/projects/bedtools/<br>MACS2 v.2.1.3: https://pypi.org/project/MACS2/2.1.3/<br>Java v.1.8: https://www.oracle.com/java/technologies/javase/javase8-archive-downloads.html<br>Juicer Tools v.1.7.5: https://github.com/aidenlab/juicer/releases<br>PLINK v.1.9: https://www.cog-genomics.org/plink2/<br>SnpEff v.5.1: https://pcingola.github.io/SnpEff/<br>R v.3.5.3: https://www.r-project.org/<br>LDSC v1.0.1: https://anaconda.org/bioconda/ldsc<br>vSampler: http://www.mulinlab.org/vsampler/

Michigan Imputation Server: https://imputationserver.sph.umich.edu/index.html#!
GraphPad Prism 8: https://www.graphpad.com

For manuscripts utilizing custom algorithms or software that are central to the research but not yet described in published literature, software must be made available to editors and reviewers. We strongly encourage code deposition in a community repository (e.g. GitHub). See the Nature Portfolio guidelines for submitting code & software for further information.

Data

Policy information about availability of data

All manuscripts must include a data availability statement. This statement should provide the following information, where applicable:

- Accession codes, unique identifiers, or web links for publicly available datasets
- A description of any restrictions on data availability
- For clinical datasets or third party data, please ensure that the statement adheres to our policy

The DNase-seq, ATAC-seq, H3K27ac ChIP-seq and Hi-C for 20 cancer type were obtained from ENCODE, Roadmap and GEO datasets, and the data sources were listed in Supplementary Data 1.
The summary GWAS statistics for each cancer type are downloaded from the GWAS catalog [https://www.ebi.ac.uk/gwas/docs/file-downloads].
The data that used for association analysis of this study are available by application from the participating consortia, including GECCO [https://www.ncbi.nlm.nih.gov/gap/] and UK Biobank [https://biobank.ctsu.ox.ac.uk/], and the dbGaP accession for GECCO program includes phs001078.v1.p1 [https://www.ncbi.nlm.nih.gov/projects/gap/cgi-bin/study.cgi?study_id=phs001078.v1.p1], phs001315.v1.p1 [https://www.ncbi.nlm.nih.gov/projects/gap/cgi-bin/study.cgi?study_id=phs001315.v1.p1] and phs001415.v1.p1 [https://www.ncbi.nlm.nih.gov/projects/gap/cgi-bin/study.cgi?study_id=phs001415.v1.p1].
The summary GWAS statistics of the BioBank Japan Project (BBJ) was available from http://jenger.riken.jp/result.
The summary GWAS statistics for the meta-analysis in CORECT, CFR1, MECC1, and GECCO can be obtained from the published research (Fredrick R Schumacher, et al. Nat Commun. 2015; 6: 7138).
The Chinese population for association analysis were described in our previous research (Tian J, et al. Cancer Res. 2020; 80(9):1804-1818). The raw sequencing data of ATAC-seq, H3K27ac ChIP-seq and RNA-seq for 10 CRC tissues have been deposited in the Gene Expression Omnibus under accession number GSE222770. Access to individual-level genotypes and multiomics sequencing from samples recruited within mainland China are subject to the policies and approvals from the Human Genetic Resource Administration, Ministry of Science and Technology (MOST) of the People's Republic China.

Research involving human participants, their data, or biological material

Policy information about studies with human participants or human data. See also policy information about sex, gender (identity/presentation), and sexual orientation and race, ethnicity and racism.

Reporting on sex and gender	The term Gender (indicated in Supplementary Table 1 as Male and Female) was used to indicate the biological attribute.
Reporting on race, ethnicity, or other socially relevant groupings	N/A
Population characteristics	The clinicopathologic information, including age, sex, tumor location, tumor pathology and TNM Staging of 10 CRC samples was collected from medical records as shown in the Supplementary Table 2. The clinicopathologic information including demographic data, age, sex, tobacco history, smoking status was collected from medical records as described in our previous research (Tian J, et al. Cancer Res. 2020 May 1;80(9):1804-1818).
Recruitment	Available tissue specimens were obtained from hospitals in Wuhan, China during 2020 to 2021 from 10 specimens from patients with primary colorectal cancer that was confirmed by histopathological examination of surgically removed tumors or biopsy specimens. The samples were collected at the time of surgery and all patients were not treated with chemotherapy or radiotherapy before tumor resection. Informed consent was obtained from each patient, and clinical information was collected from medical records.<br>The phase ? of case-control study in Chinese population recruited 1,524 CRC cases and 1,522 controls from cancer hospital of Chinese Academy of Medical Sciences in Beijing, China. The phase ? consisted of 4,500 cases and 8,500 controls from Tongji Hospital of Huazhong University of Science and Technology (HUST), Wuhan, China. All controls were cancer-free individuals selected from a community nutritional survey when patients were recruited and matched to the cases by gender and age (± 5 years). Written informed consent was obtained from each subject and the study was conducted under the approval of the participating hospitals.
Ethics oversight	CRC tissues acquisition was approved by the Ethics Committee of Tongji Hospital, Tongji Medical College of Huazhong University of Science and Technology (HUST).<br>The phase ? of case-control study was approved by the Ethics Committee of Cancer Institute and Hospital. The phase ? of case-control study was approved by the Ethics Committee of Tongji Hospital, Tongji Medical College of Huazhong University of Science and Technology (HUST).

Note that full information on the approval of the study protocol must also be provided in the manuscript.

Field-specific reporting

Please select the one below that is the best fit for your research. If you are not sure, read the appropriate sections before making your selection.

☒ Life sciences ☐ Behavioural & social sciences ☐ Ecological, evolutionary & environmental sciences

Life sciences study design

All studies must disclose on these points even when the disclosure is negative.

Sample size

Sample size was set based on the availability of CRC tissue collected. 10 available tissue specimens were obtained from the hospitals in Wuhan, China during 2020 to 2021 for ATAC-seq, H3K27ac ChIP-seq and RNA-seq and all patients were not treated with chemotherapy or radiotherapy before tumor resection. All available CRC and controls samples from collaborators were used and the sample size in this study was sufficient for two-stage case-control study. The phase ? recruited 1,524 CRC cases and 1,522 controls; The phase ? consisted of 4,500 cases and 8,500 controls. Given a disease odds ratio of 1.3 for the case group in comparison to the control group, using a significance level of α = 0.05, both stages of the two-phase case-control analysis demonstrate statistical power surpassing 0.8 (using Power and Sample Size Calculations).

Data exclusions

Following criteria were used to exclude SNPs in the imputation process: (1) imputation quality < 0.4; (2) minor allele frequency (MAF) < 1%; (3) deviating from the Hardy-Weinberg equilibrium (P < 1 × 10-6); (4) missing call frequencies > 0.02. (5) mapping to locations on sex chromosome.

Replication

Experiments in the article were reliably reproduced, replication were described in the figure legends.

Randomization

For the case-control study, randomization is not applicable as this is not a therapeutic trial and there is no intervention in this study. For the animal study, the nude mice were randomly assigned and divided into a control group and an experimental group, with 5 mice in each group.

Blinding

For the case-control study, investigators were not blinded to group allocation during data collection and/or analysis this study because the study was not designed to be any intervention or treatment for the patients. For TaqMan SNP genotyping, samples from cases and controls were randomly allocated on the same 384-well plate, ensuring that both cases and controls were tested under the same platform and experimental conditions. The experimental operators also employed blinding procedures to ensure that the specific distribution of samples was not disclosed.

Reporting for specific materials, systems and methods

We require information from authors about some types of materials, experimental systems and methods used in many studies. Here, indicate whether each material, system or method listed is relevant to your study. If you are not sure if a list item applies to your research, read the appropriate section before selecting a response.

Materials & experimental systems

n/a	Involved in the study	
☐	☒	Antibodies
☐	☒	Eukaryotic cell lines
☒	☐	Palaeontology and archaeology
☐	☒	Animals and other organisms
☒	☐	Clinical data
☒	☐	Dual use research of concern
☒	☐	Plants

Methods

n/a	Involved in the study	
☐	☒	ChIP-seq
☒	☐	Flow cytometry
☒	☐	MRI-based neuroimaging

Antibodies

Antibodies used

Primary antibodies:
Anti-H3K27ac antibodies, Rabbit polyclonal (Supplier: Abcam; Cat.: ab4729), 10μg for ChIP-seq;
Anti-IgG antibodies, Rabbit monoclonal (Supplier: Santa Cruz; Cat.: sc-66931), 10μg for ChIP-qPCR;
Anti-ZEB1 antibodies, Rabbit polyclonal (Supplier: Abcam; Cat.: ab155249), 10μg for ChIP-qPCR, 0.1μg, 0.2μg and 0.4μg for super-shift EMSAs;
Anti-PREX1 antibodies, Rabbit monoclonal (Supplier: Cell Signaling Technology; Cat.: 13168), 1:1000 dilution for Western blot;
Anti-CSE1L antibodies, Rabbit polyclonal (Supplier: Proteintech; Cat.: 22219-1-AP), 1:1000 dilution for Western blot;
Anti-STAU1 antibodies, Rabbit polyclonal (Supplier: Proteintech; Cat.: 14225-1-AP), 1:1000 dilution for Western blot, 5μg for RIP-seq;
Anti-GAPDH antibodies, Mouse monoclonal (Supplier: Proteintech; Cat.: 60004-1-lg), 1:1000 dilution for Western blot.
Secondary antibodies:
Goat Anti-Rabbit IgG secondary antibodies, HRP-conjugated; polyclonal, Proteintech; Cat.: SA00001-2, 1:5000 dilution for Western blot;
Goat Anti-Mouse IgG secondary antibodies, HRP-conjugated; polyclonal, Proteintech; Cat.: SA00001-1, 1:5000 dilution for Western blot.

Validation

All antibodies are commercially available. The validation statements are available on the manufacture's websites.
The anti-H3K27ac antibodies were suitable for ICC/IF, WB, IHC-P, ChIP, PepArr, and were reported to have undergone validation through reliable research, resulting in consistent and reproducible outcomes in each batch.
The anti-IgG antibodies were sourced as rabbit polyclonal IgG, and the validation were supported by other research studies.
The anti-PREX1 antibodies were suitable for WB, IP, IHC, ChIP, IF, etc., and were validated by other research.

The anti-CSE1L antibodies were suitable for WB, IP, IHC, IF, ELISA, and showed reactivity with human, mouse samples.
The anti-STAU1 antibodies were suitable for WB, RIP, IP, IF, ELISA, and shows reactivity with human, mouse, rat samples.
The anti-GAPDH antibodies were suitable for WB, IP, IHC, IF, FC, CoIP, ChIP, Cell treatment, ELISA, and shows reactivity with human, mouse, rat, yeast, plant, zebrafish samples.
The anti-ZEB1 antibodies were suitable for ChIP, IP, WB, ICC/IF, and were validated by other research.

Eukaryotic cell lines

Policy information about cell lines and Sex and Gender in Research

Cell line source(s)	Human CRC cell lines SW480 and HCT116 cells were obtained from the China Center for Type Culture Collection (Wuhan, China).
Authentication	All cell lines were authenticated using STR profiling.
Mycoplasma contamination	All cell lines were tested to be mycoplasma negative.
Commonly misidentified lines (See ICLAC register)	No cell lines used in this study were found in the database of commonly misidentified cell lines that is maintained by ICLAC and NCBI Biosample.

Animals and other research organisms

Policy information about studies involving animals; ARRIVE guidelines recommended for reporting animal research, and Sex and Gender in Research

Laboratory animals	Female BALB/c nude mice, aged 4-5 weeks, were used for xenograft growth of CRC cells and were purchased from the Vital River Laboratory Animal Technology (Beijing, China). All mice maintained in the specific pathogen-free (SPF) room under controlled temperature (23 ± 3?), and humidity (40-60%) conditions with 12/12h light/ dark cycle with food and water provided ad libitum.
Wild animals	The study did not involve in Wild animals.
Reporting on sex	Only female BALB/c nude mice aged 4-5 weeks were used at the time of experiments. Sample sizes for mouse experiments were empirically determined, and mice were randomly allocated to the control or experimental groups.
Field-collected samples	The study did not involve in samples collected from the field.
Ethics oversight	All experimental procedures were performed in accordance with the relevant institutional and national guidelines and approved by the experimental animal center of Wuhan university.

Note that full information on the approval of the study protocol must also be provided in the manuscript.

Plants

Seed stocks	N/A
Novel plant genotypes	N/A
Authentication	N/A

ChIP-seq

Data deposition

☒ Confirm that both raw and final processed data have been deposited in a public database such as GEO.

☒ Confirm that you have deposited or provided access to graph files (e.g. BED files) for the called peaks.

Data access links *May remain private before publication.*	To review GEO accession GSE222770:<br>May remain private before publication. Go to https://www.ncbi.nlm.nih.gov/geo/query/acc.cgi?acc= GSE222770
Files in database submission	ATAC-seq:<br>CRC_C1.R1.fq.gz<br>CRC_C1.R2.fq.gz<br>CRC_C2.R1.fq.gz<br>CRC_C2.R2.fq.gz<br>CRC_C3.R1.fq.gz<br>CRC_C3.R2.fq.gz<br>CRC_C4.R1.fq.gz<br>CRC_C4.R2.fq.gz<br>CRC_C5.R1.fq.gz<br>CRC_C5.R2.fq.gz

CRC_C6.R1.fq.gz
CRC_C6.R2.fq.gz
CRC_C7.R1.fq.gz
CRC_C7.R2.fq.gz
CRC_C8.R1.fq.gz
CRC_C8.R2.fq.gz
CRC_C9.R1.fq.gz
CRC_C9.R2.fq.gz
CRC_C10.R1.fq.gz
CRC_C10.R2.fq.gz
H3K27ac ChIP-seq:
CRC_C1_1.fq.gz
CRC_C1_2.fq.gz
CRC_C2_1.fq.gz
CRC_C2_2.fq.gz
CRC_C3_1.fq.gz
CRC_C3_2.fq.gz
CRC_C4_1.fq.gz
CRC_C4_2.fq.gz
CRC_C5_1.fq.gz
CRC_C5_2.fq.gz
CRC_C6_1.fq.gz
CRC_C6_2.fq.gz
CRC_C7_1.fq.gz
CRC_C7_2.fq.gz
CRC_C8_1.fq.gz
CRC_C8_2.fq.gz
CRC_C9_1.fq.gz
CRC_C9_2.fq.gz
CRC_C10_1.fq.gz
CRC_C10_2.fq.gz
RNA-seq:
CRC_C1.filterGC.R1.fq.gz
CRC_C1.filterGC.R2.fq.gz
CRC_C2.filterGC.R1.fq.gz
CRC_C2.filterGC.R2.fq.gz
CRC_C3.filterGC.R1.fq.gz
CRC_C3.filterGC.R2.fq.gz
CRC_C4.filterGC.R1.fq.gz
CRC_C4.filterGC.R2.fq.gz
CRC_C5.filterGC.R1.fq.gz
CRC_C5.filterGC.R2.fq.gz
CRC_C6.filterGC.R1.fq.gz
CRC_C6.filterGC.R2.fq.gz
CRC_C7.filterGC.R1.fq.gz
CRC_C7.filterGC.R2.fq.gz
CRC_C8.filterGC.R1.fq.gz
CRC_C8.filterGC.R2.fq.gz
CRC_C9.filterGC.R1.fq.gz
CRC_C9.filterGC.R2.fq.gz
CRC_C10.filterGC.R1.fq.gz
CRC_C10.filterGC.R2.fq.gz

Genome browser session
(e.g. UCSC)

https://genome.ucsc.edu/cgi-bin/hgTracks?
db=hg38&lastVirtModeType=default&lastVirtModeExtraState=&virtModeType=default&virtMode=0&nonVirtPosition=&position=chr2%3A25160915%2D25168903&hgsid=1628532837_g3RRXlELMzZ8acsFJkSUSwSShdgq

Methodology

Replicates

Each tumor sample was subjected to a single round of sequencing for ATAC-seq, ChIP-seq, and RNA-seq.

Sequencing depth

Sequencing was carried out on Novaseq 6000 sequencer (Illumina) with PE150 model by SeqHealth
#Sample_name: Unique mapped reads; Total reads
ATAC-seq_CRC_C1:76809136 94590074
ATAC-seq_CRC_C2:80138070 96697094
ATAC-seq_CRC_C3:73383257; 94059220
ATAC-seq_CRC_C4:74507745; 91224564
ATAC-seq_CRC_C5:81328564; 97810930
ATAC-seq_CRC_C6:76299692; 93412632
ATAC-seq_CRC_C7:73237113; 90019500
ATAC-seq_CRC_C8:64360796; 80150172
ATAC-seq_CRC_C9: 69993686; 87096828
ATAC-seq_CRC_C10: 70161266; 87323122
H3K27AC CHIP-seq_CRC_C1: 54361612; 67826282
H3K27AC CHIP-seq_CRC_C2: 52606734; 67601966

H3K27AC CHIP-seq_CRC_C3: 50456627; 66154468
H3K27AC CHIP-seq_CRC_C4: 54845589; 70940342
H3K27AC CHIP-seq_CRC_C5: 58171326; 73005508
H3K27AC CHIP-seq_CRC_C6: 58290627; 65260434
H3K27AC CHIP-seq_CRC_C7: 60710523; 68009404
H3K27AC CHIP-seq_CRC_C8: 55266230; 62317456
H3K27AC CHIP-seq_CRC_C9: 58911390; 76121380
H3K27AC CHIP-seq_CRC_C10: 52898084; 66004214
RNA-seq_CRC_C1: 63806103; 71368610
RNA-seq_CRC_C2: 51280751; 58489854
RNA-seq_CRC_C3: 54230261; 61734028
RNA-seq_CRC_C4: 57993521; 67757144
RNA-seq_CRC_C5: 61255490; 68172970
RNA-seq_CRC_C6: 62379299; 71382996
RNA-seq_CRC_C7: 30394344; 37362256
RNA-seq_CRC_C8: 7726519; 13264286
RNA-seq_CRC_C9: 55752952; 68339388
RNA-seq_CRC_C10: 46045996; 52456928

Antibodies

Anti-H3K27ac antibodies, Rabbit polyclonal (Supplier: Abcam; Cat.: ab4729)
Anti-IgG antibodies, Rabbit monoclonal (Supplier: Santa Cruz; Cat.: sc-66931)

Peak calling parameters

MACS2 was used to identify peaks.

Data quality

Raw sequencing reads were examined using FastQC (http://www.bioinformatics.babraham.ac.uk/projects/fastqc/). Adaptor and low-quality bases were removed using fastp. The cutoff for peaks: p-value < le-4.

Software

FastQC, fastp, Bowtie2, MACS2, IGV, BEDTools, Deeptools
